# Holding Sweden hostage: firearm-related violence

**DOI:** 10.1080/20961790.2019.1570665

**Published:** 2019-03-19

**Authors:** Ardavan Khoshnood

**Affiliations:** a Department of Criminology, Malmö University, Malmö, Sweden;; b Department of Clinical Sciences, Faculty of Medicine, Lund University, Lund, Sweden;; c Department of Emergency and Internal Medicine, Skane University Hospital, Lund, Sweden

**Keywords:** Firearm violence, Sweden, homicide, deadly violence, criminal networks, gun laws

## Abstract

Swedish as well as foreign publications have reported a sharp increase in firearm-related violence in Sweden. None of these publications, however, combined official statistics from the Swedish police, the National Council for Crime Prevention (NCCP), and the National Board of Health and Welfare (NBHW), to study firearm-related violence in the last 2 years: 2016–2017. The results of this study show that firearm-related violence in Sweden has greatly increased compared to other Scandinavian countries, especially in recent years. This is probably the reason for the increase in the rate of deadly violence. Further, the increasing number of gangs and criminal networks, as well as the high inflow of illegal firearms to Sweden, is believed to have contributed to the disturbing increase of firearm-related violence in the country. Although Sweden is one of the most democratic and freest countries in the world, and has some of the world’s strictest gun laws, the country still faces significant firearm-related violence. This study not only reveals the increasing rate of firearm-related violence in Sweden, but also shows that Sweden is in dire need of additional policies to combat the illegal flow of firearms and to curb gang criminality.

## Background

Recently published international reports state that lethal and non-lethal firearm-related violence in Sweden has been increasing; and that Sweden has one of the highest rates of shootings and consequently the highest level of firearm-related violence in Western Europe [1,2]. Swedish scholars have reported the same unsettling findings [3–7]. A Swedish register-based study recently showed that Sweden has the highest rate of lethal and non-lethal firearm-related violence among youths aged between 15 and 29 years in Western Europe [8].

Although there are no studies or reports stating the contrary, many publications are either focused on a specific Swedish geographical location [3–5], have only used one source of statistics [2], have not properly cited the used references for their findings [1], or are focused on a timeframe that is capped at 2015 [8].

The Small Arms Survey [2] writes in its latest report that Sweden, second to Israel, has had the highest increase in the proportion of firearm-related homicides from 2011 to 2016 compared to the period between 2005 and 2010. Sweden has one of the most regulated weapon laws in the Western hemisphere [3], which is the reason such claims from the above reports and studies must be evaluated and considered.

Based on official statistics from three leading sectors in Sweden, this short research article, aims to evaluate for the first time, whether firearm-related violence in Sweden between the years 2016 and 2017 indeed increased, and what the main causes may have been if it had. A statistical comparison with other Scandinavian countries (Norway, Finland, and Denmark) will also be conducted.

## A clear increase in firearm-related violence

### Data sources

The three most important sectors that gather statistics on firearm-related violence in Sweden are the Swedish police (http://www.polisen.se), the National Council for Crime Prevention (NCCP; http://www.bra.se), and the National Board of Health and Welfare (NBHW; http://www.sos.se). These three sectors’ statistics and publications on deadly and firearm-related violence were retrieved and analyzed by comparing statistical rates and figures. The use of multiple and different official sources provides a comprehensive image of firearm-related violence in Sweden, thus diminishing the risk of selection bias.

This study uses the national official statistics of other Scandinavian countries. In cases where this was not possible, statistics from the World Health Organization (https://www.who.int/healthinfo/en/) or Eurostat (https://ec.europa.eu/eurostat/web/main/home) were used.

One of the limitations of this study is that complete statistics from Denmark are lacking. Furthermore, some of the statistics used for all the countries including Sweden, may have been altered after the publication of this article. However, this alteration of statistics or the complete statistics from Denmark is not believed to deviate significantly from the figures presented.

### Deadly violence

The rate of deadly violence in Sweden has historically been low and stable [9]. In recent years, however, there have been indications of an increase in deadly violence. The NCCP [10] defines deadly violence as homicide, assault with a lethal outcome, and child homicide. This is also the definition used in the present study since, among others, data from the NCCP were used.

**Figure 1.**
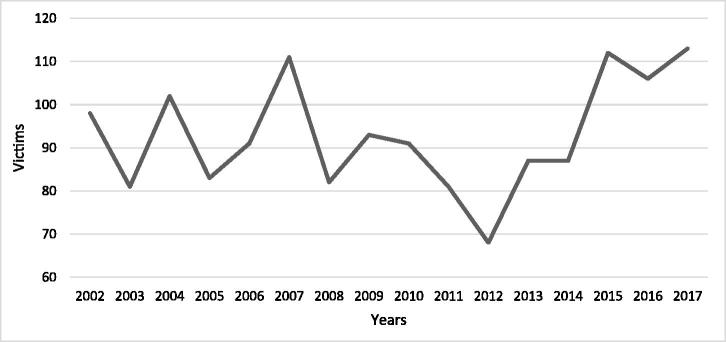
Number of individuals killed in Sweden between 2002 and 2017. Data from the National Council for Crime Prevention (NCCP) shows that 2012 had the lowest rate of deadly violence (*n* = 68), whereas 2017 had the highest (*n* = 113).

A new report from the NCCP [11] states that the number of deaths increased from 106 in 2016 to 113 in 2017. Five of the deaths in 2017 were related to the Stockholm terrorist attack in April 2017. Nonetheless, the number of victims of deadly violence increased (Figure 1): in 2017, Sweden had a homicide rate of 1.12 per 100 000 inhabitants.

### Firearm-related violence

**Figure 2.**
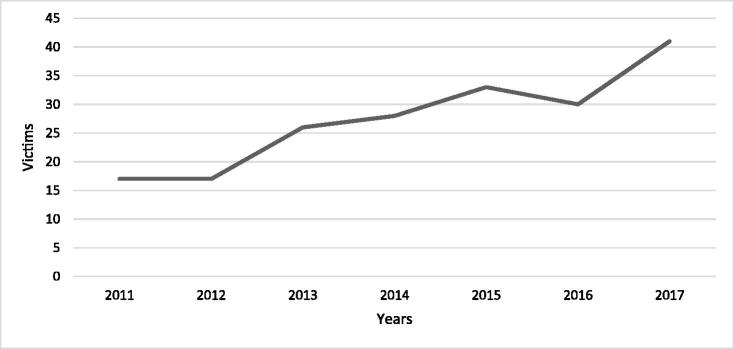
Number of firearm-related homicides in Sweden from 2011 to 2017. Data from the National Council for Crime Prevention (NCCP) show that 2011 and 2012 had the lowest number of firearm-related homicides (*n* = 17), whereas 2017, according to data from the Swedish Police, had the highest (*n* = 41) rate of firearm-related homicide.

In a report on deadly violence, the NCCP writes that a firearm was used in close to 20% of all cases of homicide in the 1990s and until 2011. The rate of firearm use for the years between 2012 and 2016, however, increased to 28% in 2016 [10]. The NCCP’s published report on deadly violence in 2017 shows that deadly violence with a firearm has greatly increased and that of the 113 individuals killed in 2017, 35% (*n* = 40) were killed by a firearm [11]. In 2017, Sweden had 0.4 firearm-related deaths per 10 000 inhabitants. Figure 2 outlines the number of individuals killed by a firearm between the years 2011 and 2017.

A recent statistical report by the Swedish police [12], shows that there were 306 shootings in Sweden in the year 2017. Of these, 135 of the victims were wounded and 41 were killed. There is, however, a slight difference between the statistics obtained from the police and those from the NCCP on whether 40 or 41 individuals were killed using firearms in 2017. An analysis of the police's statistics shows that in 2017, Sweden had on average close to six shootings each week with almost four individuals being injured or killed on a weekly basis. Sweden’s total rate of shootings per 100 000 inhabitants was 2.55 in 2017.

**Figure 3.**
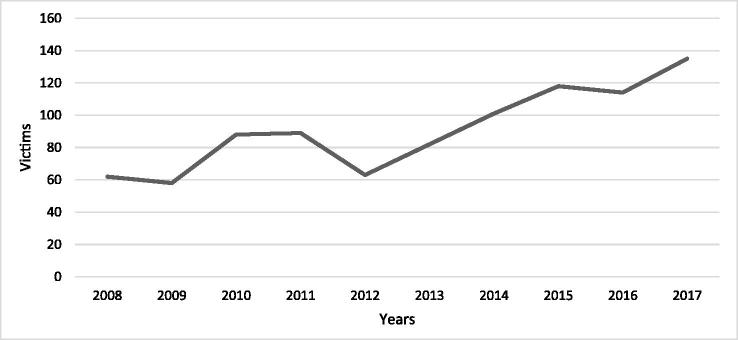
Number of individuals treated at Swedish hospitals for firearm-related injuries between 2008 and 2017. Data from the National Board of Health and Welfare (NBHW) show that 2008 had the lowest number individuals being hospitalized for a firearm-related injury (*n* = 62), whereas 2017 had the highest (*n* = 135).

Statistics from the NBHW shows that the number of individuals in Sweden injured by a firearm has greatly increased since 2009. Between 2012 and 2017, the number of individuals that were injured by a firearm increased by 50% [13]. Figure 3 outlines the number of individuals being treated at Swedish hospitals for firearm-related injuries.

### A comparison of Sweden to other Scandinavian countries

**Figure 4.**
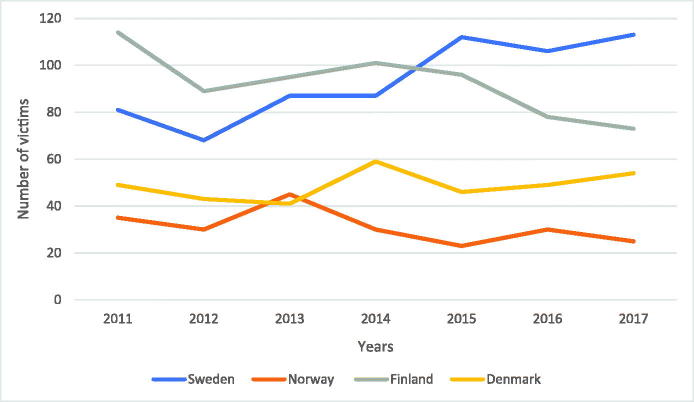
Comparison of deadly violence between Sweden, Norway, Finland, and Denmark. A comparison of deadly violence between the Scandinavian countries shows that Sweden has had the highest rate of deadly violence since 2015.

**Figure 5.**
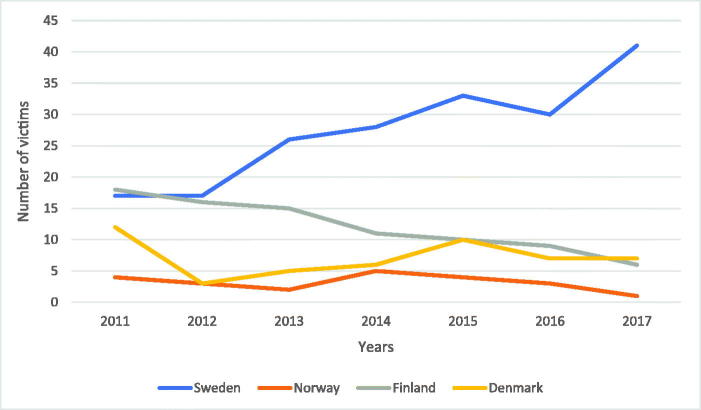
Comparison of firearm-related homicide between Sweden, Norway, Finland, and Denmark. A comparison of firearm-related homicide between the Scandinavian countries shows that Sweden has had a great increase in firearm-related homicides since 2012, significantly bypassing the other Scandinavian countries.

A comparison of Sweden to other Scandinavian countries both in regard to deadly violence (Figure 4) and firearm-related homicide (Figure 5) shows that Norway had a total deadly violence rate of 212 victims between 2011 and 2017. During the same period, a firearm was used in 22 homicides. In 2017, Norway had 25 victims of homicide, and of these, only one was killed by a firearm [14].

Finland had a total homicide rate of 646 [15] between 2011 and 2017. A firearm was used in 85 of the homicides [16]. In 2017, Finland witnessed an all-time low homicide rate with 74 individuals being killed, six of which were killed by a firearm [15,16].

Statistics on homicide in Denmark are unfortunately difficult to find. In the present study, official statistics from suspected homicides reported to the Danish Police have been used, showing a total of 341 homicides between 2011 and 2017 [17]. In 2017, Denmark yielded one of its worst homicide statistics when 54 individuals were killed.

Statistics from the World Health Organization [18] show that there were 36 firearm-related homicides in Denmark between 2011 and 2015. There are no available statistics for 2016 and 2017. Therefore, an average was calculated from the previous reported years in order to determine a likely rate of firearm-related homicides for 2016 and 2017 ((36/5) × 2 ≈ 14). The calculated rate of firearm-related homicide from 2011 to 2017 was 50 shootings.

**Figure 6.**
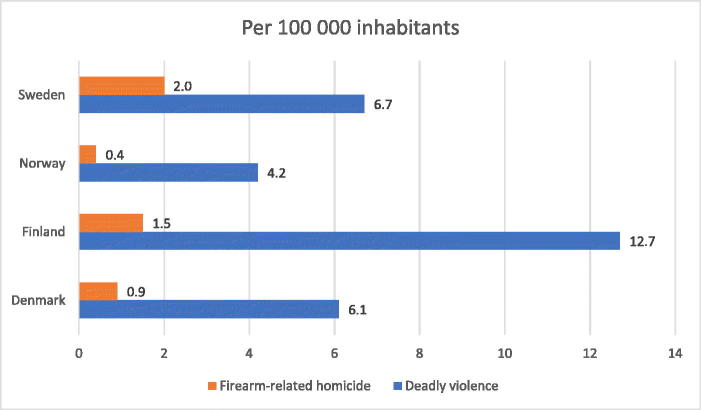
Comparison of deadly violence and firearm-related homicide between Sweden, Norway, Finland, and Denmark per 100 000 inhabitants*. Although Finland has the highest rate of deadly violence among the Scandinavian countries per 100 000 inhabitants, Sweden has the highest rate of firearm-related homicides; * Calculation of per capita is based on an average population rate of the respective country, during the studied period (2011–2017). Population data were extracted from the Eurostat database.

Figure 6 outlines both the deadly and firearm-related homicides in Scandinavian countries from 2011 to 2017, presented as per 100 000 inhabitants.

## Discussion

Statistics from the Swedish police, the NCCP, and the NBHW clearly show that the rate of firearm-related violence, including firearm-related homicides, has greatly and significantly increased in Sweden over the last decade, and not least from 2012 to 2017. This is probably the reason Sweden is also witnessing an increase in deadly violence.

International reports [1, 2], the Swedish police [12,19], and Swedish scholars [3–6,20,21] agree that the main cause for the increase in the rate of firearm-related violence is the presence of many gangs and criminal networks in Sweden.

Although gangs and criminal networks have always existed in Sweden, street gangs flourished in the late 1990s and are today considered to be one of the main security problems in the country [22–24]. Swedish gangs and foremost criminal networks have not only continued to increase, but they have also become bolder and more violent as can be seen in their use of firearms and explosive devices as their modus operandi [3,6].

Another very important source of the increase of firearm-related violence in Sweden is the easy access to illegal firearms. Although Sweden was, for decades, shielded from firearm-related violence, mostly because of its restrictive gun laws, the easy access to illegal firearms, in addition to the many gangs and criminal networks in the country, is the main reason for the disturbing increase in the country’s rate of firearm-related violence. According to police reports, there has been a high inflow of illegal weapons into Sweden from the western Balkans [12].

Sweden’s serious problem regarding illegal firearms and the many cases of shootings despite the country’s strict and restrictive gun laws, poses a new challenge for Sweden’s policy makers. Sweden’s restrictive gun laws are without a doubt the reason the country has been spared from school shootings and other types of mass shootings. These laws, however, have not succeeded in stopping the inflow and use of illegal firearms. Other countries should also be aware of this phenomenon. Restrictive gun laws must be combined with other policies and measures to fully curb firearm-related violence. At the same time, new and relatively more powerful policies are needed to combat gang criminality and gang violence.

To combat the inflow of illegal firearms, Sweden’s policy makers should address three main problems: (1) the Swedish police force must increase their collaboration with the police forces of different countries in the Balkans from where most of the weapons to Sweden are smuggled; (2) the government must introduce stricter laws regarding the smuggling of weapons. These strict laws will partly interfere with the individuals’ criminal act by incarcerating the criminals, and partly allow the police and customs to use advanced surveillance methods—not otherwise allowed for crimes warranting a short period of imprisonment—to combat gangs and criminal networks; and (3) customs must be given additional authority to maintain more effective and viable border control.

Regarding gang criminality and criminal networks, and the subsequent firearm-related violence, the situation is quite desperate in Sweden. The government and the police have significantly disrupted these criminal organizations. A stricter weapon law has been approved and implemented by the government, and the police and their intelligence unit have focused greatly on these gangs and networks. These actions may have a positive effect. However, one of the serious problems is that the participants in these criminal gangs and networks are young individuals who are promptly replaced by others whenever they get killed. This inflow of young criminals must be stopped and the way to do so is multifactorial.

Acute measures must be taken to disrupt these criminal organizations and stop the cycle of firearm violence which is holding Sweden hostage. The most violent criminals must be apprehended, and to do so, the police need both more monetary and personnel resources, as well as new laws and regulations such as the so called “stop-and-search-zones” where the police in specific zones can stop and search every single individual without probable cause as is otherwise needed. Another regulation of interest would be the so called “no-access-zones”, which prohibit individuals from specific zones either indefinitely or during certain hours.

In the long run, however, the police, schools, child and adolescent psychiatry, and social services require additional funds and resources. These institutions’ crime prevention effects must become more focused and more efficient, which is why a comprehensive education program in crime prevention is needed for these institutions. It is otherwise impossible for Sweden to stop criminal gangs and networks from recruiting youths, and consequently, maintaining the cycle of violence.

## References

[1] Savona E, Mancuso M Fighting illicit firearms trafficking routes and actors at European Level. Milano: Transcrime–Università Cattolica del Sacro Cuore; 2017.

[2] McEvoy C, Hideg G Global Violent Deaths 2017 – Time to Decide. Gonnet: Small Arms Survey; 2017.

[3] Khoshnood A The increase of firearm-related violence in Sweden. For Sci Res. 2017;2:158–160. 10.1080/20961790.2017.1314896 PMC6197136 30483635

[4] Khoshnood A, Väfors Fritz M Offender characteristics: a study of 23 violent offenders in Sweden. Deviant Behav. 2017;38:141–153.

[5] Sturup J, Rostami A, Gerell M, et al. Near-repeat shootings in contemporary Sweden 2011 to 2015. Secur J. 2018;31:73–92.

[6] Khoshnood A Firearm-related violence in Sweden – A systematic review. Aggress Viol Behav. 2018;42:43–51.

[7] Granath S, Sturup J Homicide clearance in Sweden 1990–2013 with special reference to firearm-perpetrated homicides. J Scand Stud Criminol Crime Prevent. 2018;19:98–112.

[8] Sturup J, Rostami A, Mondani H, et al. Increased gun violence among young males in Sweden: a Descriptive National Survey and International Comparison. Eur J Criminal Policy Res. 2018.

[9] Granath S, Kivivuori J, Ganpat S, et al. Homicide in Finland, the Netherlands and Sweden – A First Study on the European Homicide Monitor Data. Västerås: The Swedish National Council for Crime Prevention, The National Research Institute of Legal Policy, The Institute for Criminal Law and Criminology at Leiden University; 2011.

[10] Brottsförebyggande rådet (NCCP) Konstaterade fall av dödligt våld En granskning av anmält dödligt våld 2016 [Detected Cases of Lethal Violence: A Review of Reported lethal Violence 2016]. Stockholm: Brottsförebyggande rådet (NCCP); 2017. Swedish.

[11] Brottsförebyggande rådet (NCCP). Konstaterade fall av dödligt våld: En granskning av anmält dödligt våld 2017 [Detected Cases of Lethal Violence: A Review of Reported lethal Violence 2017]. Swedish. Stockholm: Brottsförebyggande rådet (NCCP); 2018. Swedish.

[12] Polisen (Swedish Police) Skjutningarna fortsatt många [Still too many Shootings] [Internet]. Stockholm: Polisen (Swedish Police); 2017 [cited 2018, April 7]. Available from: https://polisen.se/aktuellt/nyheter-2016-2017/2017/december/skjutningarna-fortsatt-manga/

[13] Pernilla Fagerström National Board of Health and Welfare. Personal Communication. February 12, 2018.

[14] Politiet (Norwegean Police) Nasjonal drapsoversikt 2017 – Drap i Norge 2008–2017 [National homicide overview 2017 – Homicide in Norway 2008–2017]. Oslo: Politiet (Norwegian Police); 2017. Norwegian.

[15] Statistics Finland Statistical Yearbook of Finland 2018. Helsinki: Statistics Finland; 2018.

[16] Statistics Finland Statistics Finland's PX-Web databases. Helsinki: Statistics Finland; 2018 [cited 2018, 20 December]. Available from: http://stat.fi/index_en.html

[17] Danmarks Statistik Straf20: anmeldte forbrydelser og sigtelser efter overtraedelsens art og anmeldte og sigtede [Sentence20: reported crimes and charges according to the nature of the violation and the reported crime and charged offender]. Copenhagen: Danmarks Statistik; 2018 [cited 2018, 19 December]. Danish. Available from: https://www.statistikbanken.dk/STRAF20

[18] World Health Organization WHO Mortality Database. Geneva: World Health Organization; 2018 [cited 2018, 21 December]. Available from: https://www.who.int/healthinfo/mortality_data/en/

[19] Polisen (Swedish Police). Myndighetsgemensam lägesbild om organiserad brottslighet – 2018-2019 [A Mutual Government Situational Awareness of Organized Crime – 2018-2019] Stockholm: Polismyndigheten (Swedish Police); 2017. Swedish.

[20] Khoshnood A, Fritz MV, Ekelund U Nineteen victims of homicide and attempted homicide in Sweden – Their injuries, cause of death, and offender relationship. Am J For Med Pathol. 2017;38:241–248. 10.1097/PAF.0000000000000325 28682802

[21] Leinfelt F, Rostami A The Stockholm gang model: PANTHER. Stockholm: Erlanders Sverige AB; 2012.

[22] Rostami A, Leinfelt F, Holgersson S An exploratory analysis of Swedish street gangs: applying the Maxson and Klein Typology to a Swedish Gang Dataset. J Contempor Criminal Justice. 2012;28:426–445.

[23] Rostami A Street-gang violence in Sweden is a growing concern. Sociol Forsk. 2018;54:365–368.

[24] Statsrådsberedningen (Government Offices of Sweden). Nationell säkerhetsstrategi [National Security Strategy]. Stockholm: Statsrådsberedningen (Government Offices of Sweden); 2017. Swedish.

